# Selective termination of the fetus in multiple pregnancies using ultrasound-guided radiofrequency ablation

**DOI:** 10.1186/s12884-021-04285-4

**Published:** 2021-12-10

**Authors:** Nan Li, Jimei Sun, Jiayan Wang, Wei Jian, Jing Lu, Yonghui Miao, Yufan Li, Fei Chen, Dunjin Chen, Xiaoqing Ye, Min Chen

**Affiliations:** 1 Department of Obstetrics and Gynecology, Department of Fetal Medicine and Prenatal Diagnosis, Key Laboratory for Major Obstetric Diseases of Guangdong Province, The Third Affiliated Hospital of Guangzhou Medical University, 63 Duobao Road, Liwan District, Guangzhou, China; 2 Department of Obstetrics and Gynecology, The First Affiliated Hospital of Xiamen University, Xiamen, China; 3 Department of Ultrasound, Guangzhou Women and Children’s Medical Center, Guangzhou Medical University, Guangzhou, China

**Keywords:** Selective termination, Ultrasound-guided radiofrequency ablation, Multifetal pregnancy reduction, Twin-to-twin transfusion syndrome, Selective fetal growth restriction, Discordant structural malformation

## Abstract

**Background:**

To evaluate the perinatal outcomes in women with selective termination using ultrasound-guided radiofrequency ablation (RFA).

**Methods:**

Complicated monochorionic (MC) twin pregnancies and multiple pregnancies with an indication for selective termination by ultrasound-guided coagulation of the umbilical cord with RFA under local anesthesia between July 2013 and Jan 2020 were reviewed. We analyzed the indications, gestational age at the time of the procedure, cycles of RFA, duration of the procedure, and perinatal outcome.

**Results:**

Three hundred and thirteen patients were treated during this period. Seven of whom were lost of follow-up. The remaining 306 cases, including 266 pairs of monochorionic diamniotic (MCDA) twins (86.93%), two pairs of monoamniotic twins (0.65%), 30 dichorionic triamniotic (DCTA) triplets (1%), and three monochorionic triamniotic (MCTA) triplets (0.98%), were analyzed. Indications included twin-to-twin transfusion syndrome (TTTS) (*n* = 91), selective fetal growth restriction (sFGR) (*n* = 83), severe discordant structural malformation (*n* = 78), multifetal pregnancy reduction (MFPR) (n = 78), twin reverse arterial perfusion sequence (TRAPS) (*n* = 19), and twin anemia-polycythemia sequence (TAPS) (*n* = 3). Upon comparison of RFA performed before and after 20 weeks, the co-twin loss rate (20.9% vs. 21.5%), the incidence of preterm premature rupture of membranes (PPROM) within 24 h (1.5% vs. 1.2%), and the median gestational age at delivery [35.93 (28–38) weeks vs. 36 (28.54–38.14) weeks] were similar (*p* > 0.05).

**Conclusions:**

RFA is a reasonable option when indicated in multiple pregnancies and complicated monochorionic pregnancies. In our experience, the overall survival rate was 78.76% with RFA in selective feticide, and early treatment increases the likelihood of survival for the remaining fetus because the fetal loss rate is similar before and after 20 weeks.

## Introduction

In the last three decades, the rapid development of assisted reproduction techniques (ARTs), including in vitro fertilization (IVF) and ovulation stimulation, led to a rapid rise of the incidence of multiple gestations, which are correlated with a remarkably higher risk of perinatal morbidity and mortality and, preterm delivery and a growing risk of maternal complications such as gestational diabetes, gestational hypertension, and postpartum hemorrhage [1]. Multiple pregnancies are associated with a fivefold increased risk of stillbirth and a sevenfold increased risk of neonatal death. They are associated with more complications of prematurity than singleton pregnancies [2]. The management of multiple pregnancies, discordant fetal anomalies and other complications is a clinical dilemma. Unlike the dichorionic (DC) twin placenta, where there is no vascular anastomosis, the shared placenta of the MC twins contain multiple vascular communications [3].

MC twin-specific complications such as twin-to-twin transfusion syndrome (TTTS), selective intrauterine growth restriction (sFGR), twin reverse arterial perfusion sequence (TRAPS), twin anemia-polycythemia sequence (TAPS), and conjoined twins are related to increased complications to these pregnancies [4]. Moreover, the incidence of discordant structural anomalies is more common in MC twins (6–8%) than in DC twins (1–2%). In some of these situations, selective termination of one of the fetuses may have to be considered to minimize the risk to the other fetus or to maximize the chance of the surviving fetus. In situations where there is a risk of death in the womb, choosing to terminate the affected twin may be beneficial to the healthy twin because it prevents the consequences of exsanguination of the healthy fetus into the deceased twin.

Different invasive procedures, including bipolar cord coagulation (BCC) [5], RFA, intrafetal laser coagulation [6], and microwave ablation (MWA) [7], have been described. The outcome and the survival rate of fetuses are the concern. In the literature now, few systematic reviews or meta-analyses are evaluating RFA and other reduction methods. One systematic review and meta-analysis of 481 cases of BCO and 320 cases of RFA in 17 studies showed that the overall survival rate of the fetus was 76.8% (67.6–87.2%; 238/310) for the RFA group and 79.1% (71.3–87.5%;362/459) for the BCO group [8]. Another systematic review reported that the overall survival rate after umbilical cord occlusion for selective feticide in complicated MC twins was 86% for RFA, 82% for BCC, and 72% for laser irradiation [9]. The purpose of this study is to presents our personal experience with using RFA for the selective fetal reduction in multiple gestations for different indications with large sample size. These data can add more information to the literature to reference clinical management and counseling for patients.

## Methods

This is a retrospective cohort study of consecutive cases treated with RFA from July 2013 to December 2020 at the Third Affiliated Hospital of Guangzhou Medical University in Guangzhou, the regional referral center for prenatal diagnosis and fetal medicine.

All fetuses were assessed with a detailed ultrasound examination before RFA. Fetal echocardiography and measurement of the cervical length were performed at the same time. We excluded patients who failed to follow up and included only patients with complete perinatal outcomes.

The indications for the procedure included sFGR, TRAPS, TAPS, discordant fetal anomalies, MFPR, and TTTS that cannot be treated by laser coagulation because of placental position or technique problems. Most of the TTTS cases were Quintero stage -III (80%), with a few cases of the stage -II (15%) and stage -IV (5%). For TRAPS cases, a growing acardiac mass similar in size to or larger than the normal twin was the main indication. A diagnosis of chorionicity was made according to the standard sonographic criteria [10, 11]. The indications and complications for intervention were based on ultrasound examinations. All patients were informed of the details of surgical procedures of RFA and the possible risks of the operation, such as miscarriage, preterm delivery, co-twin demise, and neurological injury in the surviving twin.

Two fetal medicine specialists performed the procedures using the same technique. The procedure was performed in an operating theatre and strictly abided by the aseptic technique. The patient was given an intravenous infusion of 1 g preventive cefazolin 1 h before surgery followed by skin antisepsis with 10% povidone-iodine, and then the operation was started. Ten milliliters of 1% lidocaine were administered locally down to the myometrium under ultrasound guidance. A small incision was made on the skin ahead of the percutaneous introduction.

A 17-gauge radiofrequency needle with a length of 15 cm and eight expandable tines at the top (MedSphere S-1500, California, USA) was used. The tines are deployed from the needle probe tip to a variable length to create a spherical space with a maximum 2-cm diameter where the thermal effects are focused.

Under ultrasound guidance, the RFA probe was inserted into the abdomen of the target twin, aiming at the region beneath the umbilical cord insertion. The device’s tines were posed within the fetal body after ascertaining the correct location. The precaution was taken to ensure that all the deployed tines were within the fetal body. A 20–40 wattage of energy generates a target temperature of 100 to 110 °C. Once the average goal temperature is reached, the device maintains the output for a defined time interval of 2 min and then shuts off to enable tissue cooling. The procedure was repeated for an additional one to two cycles until fetal bradycardia and cessation of cord blood flow were confirmed using power color and pulsed Doppler ultrasound. It is critical to keep the device away from the membranes and uterine wall to avoid thermal injury. Before the device is removed, the tines should be retracted [12].

The middle cerebral artery peak systolic velocity (MCA-PSV) was assessed within 24 h after the procedure to detect fetal anemia in the surviving twin. All women were discharged 48 h after surgery. Ultrasound examination was performed 1 week after the procedure and then every 2 weeks. Each scan involved evaluation of the fetal biometry and middle cerebral artery peak systolic velocity (MCA-PSV) of and utilized umbilical artery Doppler assessment and ductus venous (DV) Doppler in the non-targeted fetus. Fetal brain magnetic resonance imaging (MRI) is a recommended but not routine practice in the third trimester to detect brain damage in non-targeted fetuses. Most patients delivered their progeny in other hospitals in South China. Pregnancy outcomes were recorded by retrieval from the database (Astraia Software Gmbh, Ismaning, Bayern, Germany) or telephone interviews. Neonatal information and pediatric outcomes were obtained by complete clinical evaluation from the referring pediatrician and direct contact with the parents. We performed a systematic literature search in PubMed, EMBASE and clinicaltrials.gov for previous RFA studies between 2008 and 2021 and compared the data with our results.

Statistical analysis was performed using SPSS version 23 (SPSS Inc., Chicago, IL, United States). Categorical variables were compared using Fisher’s exact test or the chi-square test, as appropriate. Continuous variables were compared using unpaired Student’s t-test or the Mann–Whitney U-test. Kaplan–Meier survival curves were plotted for the time interval from RFA to delivery to compare outcomes between the RFA ≤ 20 weeks and > 20 weeks groups. The log-rank test was used to determine whether there was any difference between the two groups. A *P* value below 0.05 was considered statistically significant. Influencing factors for fetal loss after RFA were studied by binary regression analysis, including the gestational age at RFA, chorionicity, RFA indications, cycles of RFA coagulation, and duration of RFA.

## Results

**Fig. 1:**
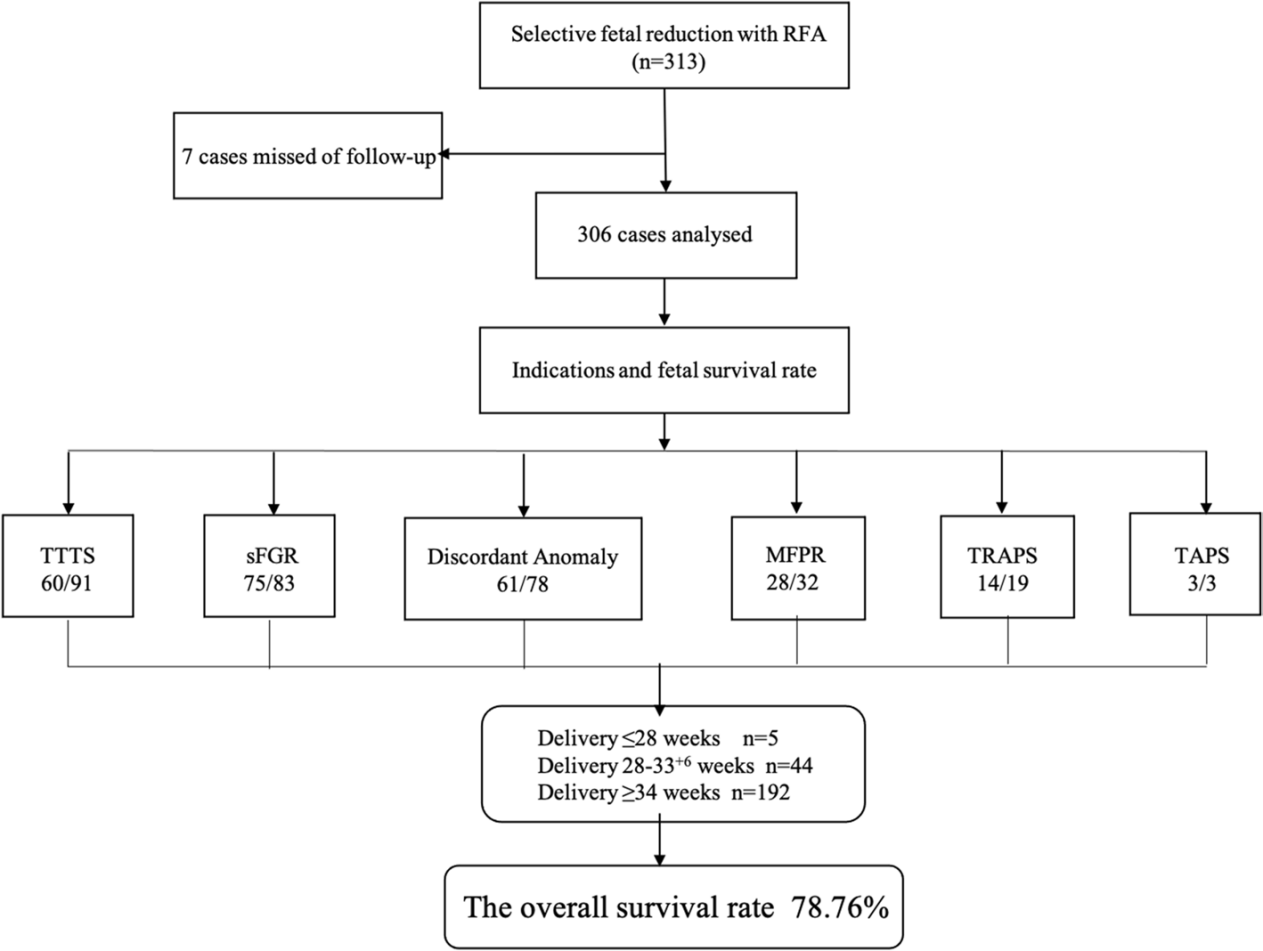
Flow chart of indications and the fetal survival rate following RFA

Three hundred and thirteen patients were treated during this period, but 7 cases were missed of follow-up. In the remained three hundred and six patients, including 266 pairs of MCDA twins, two sets of MA twins, 30 sets of DCTA triplets, and eight sets of MCTA triplets, the median gestational age of the fetuses at the time of the procedure was 20.57 (17.82–23.29) weeks. The mean maternal age was 32.71 ± 6.07 years, and the mean birth weight was 2570 ± 736 g. Indications for reduction included severe discordant structural malformation in one twin (*n* = 78), TTTS (*n* = 91), sFGR (*n* = 83), MFPR (*n* = 32), TRAPS (*n* = 19) and TAPS (n = 3). Figure 1 shows the indications and fetal survival rate following RFA. The demographics and obstetric characteristics are described in Table 1.

**Table 1:** The characteristics of cases that underwent selective termination with RFA before and after 20 weeks of gestational age

Characteristics	Total	GA at RFA ≤ 20w	GA at RFA>20w
**Number of pregnancies(n,%)**	306	134 (43.79)	172 (56.21)
**Maternal age (years)**	32.71 ± 6.07	31 (28–34.25)	30 (27–33)
**Median gestational weeks at RFA**	20.57 (17.82–23.29)	17.43 (16.39–18.78)	23 (21.18–24.29)
**Gestational age at delivery (weeks)**	36.23 ± 3.23	35.93 (28–38)	36 (28.54–38.14)
**chorionicity (n,%)**			
MCMA twin	2 (0.65)	0 (0)	2 (0.65)
MCDA twin	266 (86.93)	99 (32.35)	167 (54.58)
MCTA triplet	8 (2.61)	7 (2.29)	1 (0.33)
DCTA triplet	30 (9.80)	28 (9.15)	2 (0.65)
**Indication for RFA(n,%)**			
TTTS	91 (29.74)	28 (9.15)	63 (20.59)
sFGR	83 (27.12)	27 (8.82)	56 (18.30)
Discordant Anomaly	78 (25.49)	37 (12.09)	41 (13.40)
MFPR	32 (10.46)	28 (9.15)	4 (1.31)
TRAPS	19 (6.21)	13 (4.25)	6 (1.96)
TAPS	3 (0.98)	1 (0.33)	2 (0.65)

*MCMA* monochorionic monoamniotic; *MCDA* monochorionic diamniotic; *MCTA* monochorionic triamniotic; *DCTA* dichorionic triamniotic; *TTTS* twin-to-twin transfusion syndrome; *sFGR* selective fetal growth restriction; *MFPR* multifetal pregnancy reduction; *TRAPS* twin reversed arterial perfusion sequence; *TAPS* twin anemia-polycythemia sequence

Seven cases was lost of follow-up

The commonly reported complications associated with RFA include preterm delivery, preterm premature rupture of membranes (PPROM), and miscarriage. In our study, seventeen intrauterine fetal deaths (IUFDs) (5.56%) occurred 24 h after the procedure, and 4 (1.31%) occurred two weeks later. The total number of cases of PPROM and miscarriage after the procedure was 37 (12.1%). In 6 cases, the couples opted for termination of pregnancy. Approximately one-half of these PPROM cases occurred more than two weeks after the operation.

The total fetal survival rate after RFA was 78.76%. SFGR had the best outcome in the cohort, with fetal loss in only 8 of 83 cases. Surgical failure was defined as a procedure that could not stop the blood flow in the umbilical cord, which occurred only once (0.33%). The case was a TRAPS in which the diameter of the acardiac mass exceeded the abdominal circumference of the unaffected fetus at 23 weeks. Blood flow in the acardiac mass could not be stopped successfully, so the woman was monitored weekly. She spontaneously delivered a 3000 g baby. The fetal demise of the healthy twin within 24 h following RFA occurred in 17 cases (5.56%) and 2 weeks after RFA in another 4 cases. Termination of pregnancy was performed in 6 cases at the couples’ request. In two cases of TTTS, subsequent examination of the surviving co-twin revealed evidence of cerebral atrophy, which was confirmed by MRI. Three cases developed mild anemia within 48 h after the procedure, but there was no obvious neurological damage at long-term follow-up.

Table 2 shows the univariable and multivariable logistic regression analysis of the potential factors affecting co-twin loss after the procedure. Compared to the co-twin alive group, the co-twin loss group was associated with the indication itself. There was no statistically significant correlation with the gestational age at the time of RFA, the chorionicity of fetuses, cycles of RFA coagulation, or the duration of RFA.

**Table 2:** Univariable and multivariable logistic regression analyses were used to confirm several possible factors affecting co-twin fetal loss after RFA

Variable	Group 1 co-twin alive ***N*** = 241	Group 2 co-twin loss ***N*** = 65	Univariate		Multivariate	
	(n,%)	(n,%)	P	OR (95% CI)	P	OR (95% CI)
**Gestational weeks at RFA**						
≤20 weeks	106 (43.9)	28 (43.1)	0.9	0.96(0.55–1.68)	0.68	1.14 (0.61–2.21)
>20 weeks	135 (56)	37 (56.9)		reference		reference
**Chorionicity**						
Monochorionic	215 (89.2)	61 (93.8)	0.77	1.14 (0.47–2.80)	0.91	0.95 (0.37–2.41)
Dichorionic	26 (10.8)	4 (6.2)		reference		reference
**Indications of RFA**						
TTTS	60 (24.9)	31 (47.7)		reference		reference
sFGR	75 (31.1)	8 (12.3)	< 0.001	2.06 (0.09–0.482)	< 0.001	0.21 (0.09–0.5)
Discordant Anomalies	61 (25.3)	17 (26.2)	0.08	0.54 (0.27–1.08)	0.07	0.53 (0.26–1.06)
MFPR	28 (11.6)	4 (6.2)	0.03	0.28 (0.09–0.86)	0.03	0.27 (0.08–0.87)
TRAPS	14 (5.8)	5 (7.7)	0.51	0.69 (0.23–2.10)	0.43	0.63 (0.2–1.98)
TAPS	3 (1.2)	0 (0)	1	N/A	1	N/A
**Cycles of RFA coagulation**						
≤2	193 (80.1)	48 (73.8)	0.28	0.70 (0.37–1.33)	0.3	0.7(0.35–1.37)
>2	48 (19.9)	17 (26.2)		reference		reference
**Duration of RFA (min)**						
≤15	169(70.1)	49(75.4)	0.41	0.77 (0.41–1.44)	0.49	1.26(0.65–2.45)
>15	72(29.5)	16(24.6)		reference		reference

*OR* odds ratio; *CI* confidence interval; *N/A* not applicable

The median gestational age at RFA was 17.43 (16.39–18.78) weeks and 23 (21.18–24.29) weeks in the groups undergoing RFA before 20 weeks and after 20 weeks, respectively (Table 1). The median gestational age when PRROM occurred in the two groups was [21.9 (15.6–26) weeks vs. 25.6 (23.8–26.7) weeks; *p* = 0.145], respectively. The mean gestational age of preterm delivery before 34 weeks was similar between the two groups (30 ± 1.6 vs. 30.7 ± 1.72; *p* = 0.142) (Table 3). There was also no difference in the median gestational age at delivery [35.93 (28–38) weeks vs. 36 (28.54–38.14) weeks; *P* = 0.253] or mean birth weight (2528 ± 776 g vs. 2605 ± 705 g; *p* = 0.438) (Table 3).

**Table 3:** Comparison of the outcomes of selective termination with RFA performed before and after 20 weeks of gestational age

	GA at RFA ≤ 20 weeks	GA at RFA>20 weeks	P value
**pregnancies after RFA**			<0.001
singleton pregnancies	101 (75.4)	164 (95.4)	
twin pregnancies	33 (24.6)	8 (4.7)	
**outcome**			0.22
Live born	106 (79.1)	135 (78.5)	
Failure	1 (0.7)	0 (0)	
Miscarriage	7 (5.2)	13 (7.6)	
IUFD	6 (4.5)	15 (8.7)	
TOP	3 (1.0)	3 (1.7)	
PPROM	11 (8.2)	6 (4.5)	
**Gestational week at PPROM**	21.9 (15.6–26)	25.6 (23.8–26.7)	0.145
(1) PPROM within 24 h	2 (1.5)	2 (1.2)	
(2)24 h ≤ PPROM≤4 weeks	4 (3.0)	4 (2.3)	
(3) PPROM after 4 weeks	5 (3.7)	0 (0)	
**Alive fetuses**			
Gestational week at delivery	35.93 (28–38)	36 (28.54–38.14)	0.253
Gestational age of preterm delivery before 34 weeks	30 ± 1.6	30.7 ± 1.72	0.142
Interval from RFA to delivery (wks)	18.5 ± 3.8	13 ± 3.9	<0.001
Birthweight of alive fetus (g)	2528 ± 776	2605 ± 705	0.438

Data are presented as n (%), median, mean ± SD

*IUFD* intrauterine fetal demise; *TOP* termination of pregnancy

**Fig. 2:**
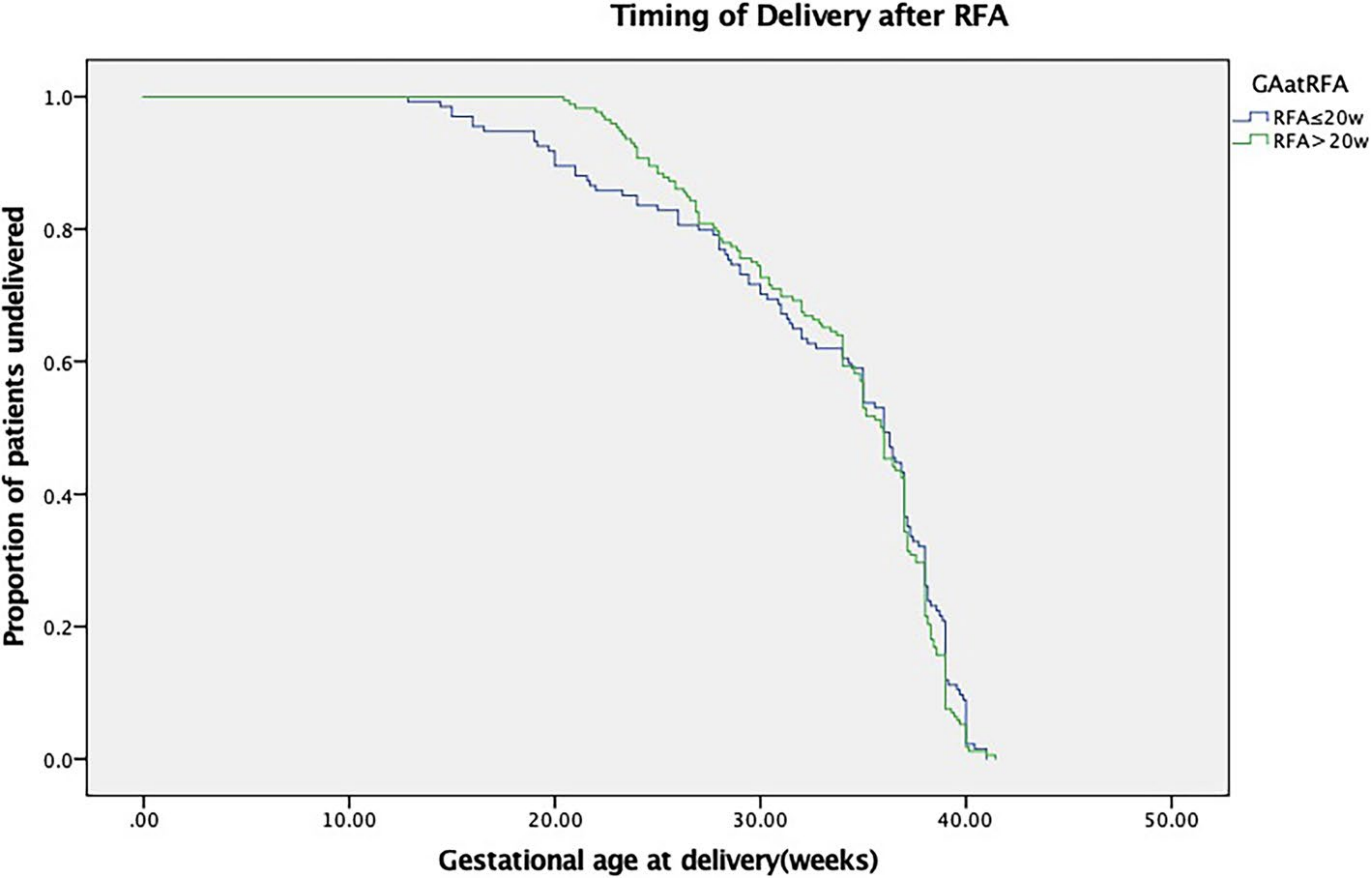
Kaplan–Meier survival curve showing the proportion of cases continuing the pregnancy after RFA was performed before and after 20 weeks of gestational age

The Kaplan–Meier survival curve showed the proportion of cases continuing the pregnancy after RFA in the two groups (Fig. 2). The two curves show that the gestational age at the time of RFA had no significant difference in the pregnancy outcome (*P* = 0.882).

Table 4 summarises the results of this current study and compares with those of 22 previous studies that have evaluated RFA. The overall survival rates, gestational age at the time of the procedure, and gestational age at delivery were reported in all studies. However, most of the studies did not specifically evaluate the birth weight, cycles of RFA coagulation, or duration of RFA. The combined data show that the mean fetal survival rate was 76.55 ± 8.01%.

**Table 4:** Review of RFA studies

Study	Case ,n	Chorionicity	The overall survival rate,%	The mean/median Gestational Age at RFA	The mean/median Gestational Age at Delivery	Birthweight(g)	Indications of RFA						Cycles of RFA coagulation		Duration of RFA (min)	
							TTTS	sFGR	DA	MFPR	TRAPS	TAPS				
current study	306	215 M 26 D	78.76	20.57 (17.82–23.29)	36.23 ± 3.23	2570 ± 736	91	83	78	32	19	3	193n ≤ 2	48n>2	169n ≤ 15	72n> 15
Wang et al. (2021) [13]	272	272 M	73.9	20.05 ± 3.41	36.34 ± 2.9	2662.48 ± 707.8	64	60	66	70	12	0	NA		NA	
Shinar et al. (2021) [14]	74	74 M	91.9	19.3 ± 4	34.5 ± 6.5	2477 ± 1016	6	9	24	0	35	0	NA		27.4 ± 15.8	
TING et al. (2021) [15]	63	61 M 2 D	73.02	17.4 (13.6–19.1)	36.2 (35–38.8)	2497(2170–2926)	12	10	17	9	13	2	NA		40n < 4	4n > 4
Rahimi-Sharbaf et al. (2021) [16]	143	143 M	71.3	21 ± 2.3	34.6 ± 3.3	NA	48	52	33	0	10	0	Usually, 2–3		NA	
Liu et al. (2021) [17]	56	56 M	73.2	20.5 ± 3.3	31.6 ± 6.5	NA	26	11	9	4	4	0	NA		NA	
Dadhwal et al. (2021) [18]	44	44 M	77.3	22.29(14–26.86)	35(32.14–37)	2138 ± 742	23	5	7	0	9	0	NA		usually< 15	
Gabby et al. (2020) [19]	36	36 M	75	19.78	36.6(23.7–41)	NA	2	10	9	0	15	0	50n < 4	9n ≥ 4	NA	
Dadhwal et al. (2019) [20]	14	14 M	71.4	24.43(16–26.57)	36(28–38)	NA	NA	NA	NA	NA	NA	NA	NA		NA	
Sun et al. (2018) [21]	183	183 M	77	19.6(17.3–22.5)	36.8 (33.2–38.5)	NA	35	53	24	36	35	0	152n ≤ 2	25n>2	6 (4–7)	
Abdel-Sattar et al. (2018) [22]	18	18 M	66.7	19.1 (16.9–25.4)	34.6 (17.4–40.1)	2857 (538–4451)	0	2	6	0	10	0	NA		NA	
Wang et al. (2017) [23]	33	M	84	20.2 ± 3.8	36.9 ± 2.6	2700(2275–3025)	6	4	10	11	2	0	NA		NA	
Peng et al. (2016) [24]	45	45 M	71.1	19.86(18.14–26.71)	31.57(22.29–40.86)	1575 (250–3400)	15	10	8	0	12	0	NA		NA	
Yinon et al. (2015) [25]	36	36 M	88.9	21.3 (17.7–24.3)	35.0 (29.8–38.0)	2405 (1606–3220)	6	19	7	0	4	0	≤3		≤12	
Kumar et al. (2014) [26]	100	82 M 18 D	78.0	17.96 (12.14–27.57	35.2(24–41)	NA	28	8	38	14	12	0	2–3		most cases ≤15	
Berg et al. (2014) [27]	7	7 M	85.7	23.0 ± 5.0	32.3 ± 5.3	NA	0	0	0	0	7	0	NA		6	
Van Den Bos et al. (2013) [28]	11	11 M	63.6	15(14–18)	34(23–38)	NA	1	2	2	1	5	0	NA		NA	
Lu et al. (2013) [29]	10	10 M	NA	15.6 (12.3–19.6)	35.9(32.4–38.6)	NA	1	2	4	0	3	0	most ≤2	little>2	NA	
Cabassa et al. (2013) [30]	7	7 M	71	17.43(13.14–23.14)	33.00	NA	0	0	0	0	7	0	1–3		≤6	
Bebbington et al. (2012) [31]	58	58 M	70.7	20.2 ± 2.2	33.0 (23.4–38.9)	NA	15	19	6	0	18	0	NA		NA	
Roman et al. (2010) [32]	20	20 M	87	20.3 (17–29)	36 (26–41)	2350 ± 1164	4	2	8	0	6	0	≤3		2	
Paramasivam et al. (2010) [33]	35	29 M 6 D	88.6	17.4 (12.71–27.57)	36(21.86–41)	NA	11	4	9	6	5	0	NA		12(median)	
Moise et al. (2008) [34]	9	9 M	66.0	19.5 (18.6–22)	36.1(26.0–39.2)	NA	3	0	6	0	0	0	NA		6	

*N/A* not applicable; *M* Monochorionic; *D* Dichorionic; *DA* Discordant Anomalies

## Discussion

Our study demonstrated that the indications of RFA were the potential risk factors for co-twin loss after RFA, while gestational age at RFA, chorionicity, cycles of RFA coagulation, and the duration of RFA were not. Other studies concluded that the number of ablation cycles was inversely associated with the fetal loss rate [15, 21]. Our study compared the fetal death rate between cases where RFA was performed before 20 weeks and after 20 weeks (26.4% vs. 27.4%, respectively). Based on these similar results, we concluded that it is best to intervene early to minimize complications.

In Table 4, the two highest fetal survival rates, 91.9% and 88.6% were reported by Shinar et al. [14] and Paramasivam et al. [33], respectively. We combined the survival data from all the included articles, and the final mean survival rate was 76.55 ± 8.01%. In the current study, the overall survival rate was 78.76%, which is consistent with the final mean survival rate of the combined studies. Our result is also in accordance with that of Gaerty et al., who performed a systematic review and meta-analysis and found that the survival rate of the RFA group was 76.8% (67.6–87.2%; 238/310) [8]. This study and previous reports provide accurate information for couples’ counselling about this procedure [15, 21].

As experience in using RFA to treat fetuses with TRAPS has increased, RFA has been considered a viable alternative to treat TRAPS [32]. One review reporting 98 registry cases on the outcomes of using RFA to treat TRAPS from 1998 to 2008 suggested that the survival rate was 80% in the overall cohort and that the mean gestational age at delivery was 36 weeks [35].

In our 19 cases of TRAPS, 14 co-twins survived. And 61 infants from 78 cases of MFPR survived. The perinatal outcomes were better in the sFGR than the TTTS cases (75/83 (90.36%) vs. 60/91 (65.93%)). Another review also concluded that pregnancies with TTTS appear to have a lower overall survival rate than pregnancies treated for other indications [8]. Compared to reduction for severe TTTS, selective reduction due to sFGR is associated with a more favorable perinatal outcome. One possible reason for this phenomenon could be that both fetuses are affected by TTTS, and the presence of polyhydramnios increases the risk of preterm delivery. Another explanation could be that the fetuses affected by TTTS had prolonged exposure to massive hemodynamic changes, contributing to worse perinatal outcomes, whereas only the smaller fetus is affected by sFGR.

Iatrogenic PPROM is another major complication. There is evidence that the use of smaller devices to enter the amniotic cavity may be associated with a lower risk of PPROM [32]. The PPROM rate risk is 25–50% when a 3.3-mm trocar of BCC is used. The main advantage of RFA over BCC is the introduction of a minor membrane defect less than 3.3 mm owing to the 17-gauge needle. As Table 3 shows, more than half of the cases (12/17) developed PPROM within 4 weeks after the RFA procedure. This complication contributes to significantly elevated rates of adverse perinatal outcomes. Selective termination in MC pregnancies has also been associated with other perioperative complications, including late miscarriage, amniotic band syndrome, chorioamnionitis, and other procedure-related fatal injuries [12]. The fetal reduction is also a better choice to reduce the maternal-fetal complications for multiple pregnancies. Chaveeva et al. reported the outcome of DC triplet pregnancies reduced to DC twins by laser ablation and concluded that fetal reduction has a lower miscarriage rate and a lower preterm birth rate of < 33 weeks’ gestation compared with expectant treatment [36]. A meta-analysis suggests that multifetal pregnancy reduction of triplet pregnancies to twin pregnancies is associated with a better pregnancy outcome than that of non-reduced triplets [37]. For the interval from RFA to delivery, because the gestational age of the two groups at delivery is similar, the interval between the operation and delivery will be longer for women who had the fetal reduction before 20 weeks of gestation.

Accurate placement of the needle under ultrasound guidance is crucial to the success of the procedure. Sometimes it is difficult to place the probe in the ideal site, especially when the fetus is facing down during the surgery, which may increase the failure rate and risk of the procedure. We rarely place the needle multiple times because we track the exact position of the needle from the initial insertion under the ultrasound guidance. After ablation, visualization of the scorched area is usually poor, making it challenging to observe blood flow [38]. For TTTS in this cohort of patients, we chose the RFA because fetoscopic laser treatment is often hindered by technical difficulties such as reduced visibility due to stained amniotic fluid or poor accessibility of some anastomoses due to placenta location or the position of fetal parts on the vascular equator [39]. In addition, the couples preferred to keep a healthy singleton than to keep babies with neurological damages in monochorionic twins. Three surviving fetuses were anemic following the procedure, and twenty-one cases were associated with fetal demise after the procedure. In MC pregnancies, the life-threatening condition of one twin can cause a severe hemodynamic imbalance, at least a 25% chance of intrauterine death of the healthy co-twin and neurological sequelae in approximately 25% of surviving twins [15]. This is assumed to be due to the exsanguination of the normal twin into the affected twin in the presence of placental vascular anastomoses [9]. These results may be due to incomplete coagulation of the cord in the affected twin to a large extent. Power color Doppler and pulsed Doppler are useful for monitoring the quality of coagulation as soon as the target fetus dies, suggesting that it is necessary to follow up cases closely using MCA Doppler [40]. Although selective feticide aims to protect the life and well-being of the co-twin, the primary concern after an intervention is the risk of intrauterine death of the co-twin. Abdel-Sattar et al. reported that 33.3% of co-twins died after the procedure [22]. The period of highest risk is within 24–48 h after the operation. In our cohort, the percentage of co-twin intrauterine deaths was 6.9% (21/306), with the most likely causes of acute transfer of intravascular volume from the co-twin to the hypotensive, dying fetus of unsuccessful or incomplete coagulation of the target vessels. Sometimes intrauterine death of the co-twin may occur late, several weeks after surgery, and the underlying reason remains unknown [40].

It has been reported that the fetus suffered thermal damage after RFA [26]. In our cohort, we found no instances of uterine or fetal heat injury. Proficient operative experience and careful preoperative assessment play a significant role in the development of complications. The studies by D’Antonio F et al. and Schou KV et al. confirmed that skills and experience could improve surgical outcomes [41] [5]. Therefore, only after sufficient professional technical training should physicians perform selective termination reduction operations.

One of the strengths is that this report reviews all RFA studies in recent years and compares them with ours. Second, the sample size of this study was relatively large, and almost every kind of complication was included, although the number of some complications (such as TAPS and TRAPS) was relatively small. One limitation of this paper is that it is a single-center retrospective study, and there may be bias in the data interpretation. Further multicenter prospective studies are needed to more effectively identify risk factors for co-twin fetal loss following RFA.

## Conclusion

In summary, RFA is a relatively safe technique for selective fetal reduction, and the indication for RFA is a risk factor of fetal loss. The survival rate of co-twins was similar when RFA was performed before and after gestational age 20 weeks. Early detection and timely treatment for MC complications is likely to increase the chance of survival for fetuses.

## Data Availability

The datasets generated and/or analyzed during the current study are available from the corresponding author on reasonable request.

## Abbreviations

MC: Monochorionic
RFA: Radiofrequency ablation
MCDA: Monochorionic diamniotic
MCTA: Monochorionic triamniotic
DCTA: Dichorionic triamniotic
TTTS: Twin-to-twin transfusion syndrome
sFGR: Selective fetal growth restriction
MFPR: Multifetal pregnancy reduction
TRAPS: Twin reversed arterial perfusion sequence
TAPS: Twin anemia-polycythemia sequence
ARTs: Reproduction techniques
IVF: In vitro fertilization
BCC: Bipolar cord coagulation
MWA: Microwave ablation
MCA-PSV: Middle cerebral artery peak systolic velocity
MRI: Magnetic resonance imaging
PPROM: Preterm premature rupture of the membranes
